# Corifollitropin Alfa for Controlled Ovarian Stimulation in Assisted Reproductive Technologies: State of the Art

**DOI:** 10.1055/s-0042-1759631

**Published:** 2023-03-06

**Authors:** Bruno Ramalho de Carvalho

**Affiliations:** 1 Bruno Ramalho Reprodução Humana, Brasília, DF, Brazil

**Keywords:** corifollitropin alfa, ovulation induction, ovarian stimulation, gnrh antagonist, assisted fertilization, alfacorifolitropina, indução de ovulação, estimulação ovariana, antagonista de gnrh, reprodução assistida

## Abstract

Physical and emotional burdens during the journey of infertile people through assisted reproductive technologies are sufficient to justify the efforts in developing patient-friendly treatment strategies. Thus, shorter duration of ovarian stimulation protocols and the need for less injections may improve adherence, prevent mistakes, and reduce financial costs. Therefore, the sustained follicle-stimulating action of corifollitropin alfa may be the most differentiating pharmacokinetic characteristic among available gonadotropins. In this paper, we gather the evidence on its use, aiming to provide the information needed for considering it as a first choice when a patient-friendly strategy is desired.

## Resumo

O desgaste físico e emocional durante a jornada de pessoas inférteis pelas tecnologias de reprodução assistida é suficiente para justificar esforços no desenvolvimento de estratégias de tratamento compassivas. Desta forma, a menor duração dos protocolos de estimulação ovariana e a necessidade de menos injeções podem melhorar a adesão, prevenir erros e reduzir custos financeiros. Portanto, a estimulação folicular sustentada da alfacorifolitropina parece ser a característica farmacocinética que melhor a diferencia das gonadotrofinas atualmente disponíveis no mercado. No presente artigo, reunimos evidências sobre seu uso, com o objetivo de fornecer as informações necessárias para considerá-la como primeira escolha quando se deseja uma estratégia amigável ao paciente.

## Introduction

The evident physical and emotional burdens during the journey of infertile people through assisted reproductive technologies (ARTs) are sufficient to justify the efforts in developing patient-friendly treatment strategies. Aiming to offer the most possible comfort to the experience, alleviating distress may also reduce the known treatment drop-out because of its psychological impact. 1

It has been already demonstrated that the shorter duration of ovarian stimulation protocols using GnRH antagonists may be associated to significantly reduced discontinuation rates. 2 Similarly, the need for less injections may improve adherence, prevent mistakes, and reduce financial costs. 3 4

Therefore, the sustained follicle-stimulating action is the most differentiating pharmacokinetic characteristic of corifollitropin alfa, when compared with any of the available recombinant follitropin (rFSH) presentations used in ovarian stimulation protocols for ART. With similar pharmacodynamic profile as rFSH, multiple follicular growth initiated by corifollitropin alfa is kept for an entire week. In other words, one adequate dose injection (100 μg for women weighting up to 60 kg, and 150 μg for those > 60 kg) replaces the first 7 daily doses of the other gonadotropins. 3 5

In addition to the simplicity of the treatment and the reduction of emotional burden is the possibility of achieving the criteria of triggering final oocyte maturation on day 8 of stimulation, dispensing daily gonadotropins from that day onwards. 3 5

In the present paper, the evidence available on corifollitropin alfa is revisited, aiming to provide the information needed for considering it as a first choice when a patient-friendly strategy is desired.

## Methods

The PubMed database was systematically searched for clinical trials, randomized controlled trials (RCTs), and meta-analyses on any ART outcome after the use of corifollitropin alfa for ovarian stimulation, published between 2012 and 2021, in the English language. Additionally, the original trials ENGAGE and ENSURE, and published post-hoc analyses of their data were included. Finally, references from the eligible papers which were of moderate or good quality were included.

## Duration of Stimulation

The duration of stimulation was the same for corifollitropin alfa and rFSH in both the ENGAGE and the ENSURE trials at any dose (median of 9 days). Also, no significant differences were found for the need of daily rFSH from day 8 onwards or discontinuation rates in both studies. 3 5 In a post-hoc analysis of the ENGAGE trial, Mardešič et al. 6 concluded that a short duration of stimulation (6 to 8 days) resulted in the same chance of achieving an ongoing pregnancy as that observed in longer cycles, regardless of the use of either corifollitropin alfa or daily rFSH.

## Number of Retrieved Oocytes, Ongoing Pregnancy Rate, and Live Birth Rate

Considered as coprimary endpoint in the ENGAGE Trial, the mean ± standard deviation (SD) number of cumulus-oocyte-complexes (COCs) retrieved after corifollitropin alfa stimulation was 13.7 ± 8.2, which was significantly higher than the correspondent 12.5 ± 6.7 for daily rFSH. According to the authors, such a finding suggests at least an equivalent pharmacological effect of both gonadotropins. 3 Similar numbers of COCs yielded per started cycle were demonstrated in the ENSURE Trial (13.3 ± 7.3 in the corifollitropin alfa group versus 10.6 ± 5.9 for daily rFSH, *p* < 0.001), 5 the TRUST trial (11.9 ± 7.2 in the first cycle using corifollitropin alfa, and 11.3 ± 7.6 in the third cycle), 7 and the PURSUE trial (10.7 ± 7.2 in the corifollitropin alfa group versus 10.3 ± 6.8 for daily rFSH, estimated difference 0.5, 95% confidence interval [CI]: 0.2–1.2). 8 Ongoing pregnancy was the primary endpoint for the ENGAGE Trial, considering the identification of fetal heart activity assessed by ultrasound at least 10 weeks after embryo transfer (coherently, confirmed live births were given the same value as endpoint). This trial pointed similar ongoing pregnancy rates per initiated cycle after follicular stimulation by corifollitropin alfa and daily rFSH (38.9 and 38.1%, respectively). 3 In the ENSURE Trial, the ongoing pregnancy rates per started cycle for the corifollitropin alfa in the 100 μg dose and the daily rFSH groups were 25.4 and 34.4%, respectively. Despite the apparent difference between regimens, it was not statistically significant. 5 Moreover, in the RCT conducted by Boostanfar et al. 8 for women aged 35 to 42 years old, live birth rates after fresh embryo transfer and the estimated cumulative live birth rates were statistically equal with corifollitropin alfa and daily rFSH in a GnRH antagonist protocol (35.6 versus 34.4%, and 43.4 versus 41.3%, respectively). In accordance, the meta-analysis of 8 randomized controlled trials, including 4,340 in vitro fertilization (IVF) cycles, found similar multiple pregnancy, ongoing pregnancy and/or live birth rates between corifollitropin alfa users and controls, whether in normal or poor responders. 9 The findings reassured the previous meta-analysis of six RCTs conducted by Pouwer et al., 10 who concluded from moderate quality evidence that the use of 150 μg of corifollitropin alfa is as efficient as daily rFSH regarding rates of ongoing pregnancies and live births. Nevertheless, a reduced live birth rate was found by them for women receiving lower doses (60 to 120 μg) of the long-acting rFSH. 10 Finally, in an individual data meta-analysis including 3 RCTs, women aged 18 to 36 years old weighting > 60 kg (ENGAGE) or ≤ 60 kg (ENSURE), or those aged 35 to 42 years old weighting ≥ 50 kg (PURSUE) using corifollitropin alfa or daily rFSH presented at least equivalent numbers of oocytes retrieved, vital pregnancy rates, ongoing pregnancy rates, and live birth rates. 11

## Other Reproductive Outcomes

In the ENGAGE Trial, the number of follicles ≥ 17 mm on the day of human chorionic gonadotropin (hCG) administration, fertilization rates, and total number of embryos and high-quality embryos were very close between the two stimulation regimens tested, and independent from adopted ART procedures. 3 Comparable numbers of follicles ≥ 11 mm in the hCG day were also seen by the ENSURE and the PURSUE investigators for both corifollitropin alfa and rFSH. 5 12 Compiling the two above-mentioned meta-analyses, comparable rates of cycle cancellation, ovarian hyperstimulation syndrome (OHSS), multiple pregnancy rate, miscarriage rate, ectopic pregnancy rate, or congenital anomalies' rate were found for corifollitropin alfa and daily gonadotropins. 9 10 Ultimately, the frequency of preterm births and the incidence of neonatal/infant adverse events were similar between protocols using corifollitropin alfa and daily rFSH in both the ENGAGE and ENSURE trials. The overall incidence of any congenital defects was 16.3 and 17.0% for corifollitropin alfa and rFSH, respectively, with comparable incidences of major malformations. 13

## Adverse Events

Corifollitropin alfa has been well-tolerated, without concerns on immunogenicity, and leading to adverse events similar to the observed with other gonadotropins, with headache and pelvic pain being often mentioned by patients. 7 Similar incidences of moderate or severe adverse events were observed between corifollitropin alfa and rFSH in the ENGAGE Trial, and rates of OHSS were statistically the same. 3 A pooled analysis of data from the ENGAGE and ENSURE trials indicated a statistically insignificant elevation of the risk of OHSS following corifollitropin alfa when compared to rFSH, with a difference of 0.5% for severe OHSS. Of note, the observed incidence of OHSS in the TRUST trial, which is supposed to be closer in line with current medical practice, was lower than those observed in the ENGAGE and ENSURE trials. 14 The post-hoc study by Griesinger et al. (2016) 15 including 2,433 women from the ENGAGE, ENSURE and TRUST trials, who received hCG for follicular maturation, which found an optimal threshold of 19 follicles ≥ 11 mm on the day of hCG to predict the occurrence of moderate or severe OHSS was interesting. Additionally, for estradiol levels on the day of hCG, the authors established the optimal threshold on 1,634 pg/mL (6,000 pmol/L), but with less prognostic value. 15 In another post hoc analysis of data from ENGAGE and PURSUE, progesterone elevation > 1.5 ng/mL in the day of the trigger was significantly less frequent among patients who underwent stimulation with corifollitropin alfa versus daily rFSH, considering patients who did not need any additional rFSH after day 8. However, if rFSH is administered from day 8 onwards, a nonsignificant trend toward premature progesterone elevation was detected, with no significant difference between corifollitropin and daily rFSH. 16 Finally, multiple pregnancies tended to be more frequent for corifollitropin alfa (+ 4.4% absolute risk increase) than for daily rFSH in the ENGAGE Trial. 3

## Flexible and Alternative Regimens

The post hoc analysis of the ENGAGE trial found no differences in ongoing pregnancy rates between women: those who received corifollitropin alfa on menstrual cycle day 2 versus day 3; who received step-down or fixed-dose of rFSH from day 8 onwards; who received rFSH on the day of the trigger or who did not; or, finally, who received hCG in the expected day versus those with a 1-day delay. 17 The administration of corifollitropin alfa on cycle day 4 has also been tested as an alternative regimen to the standard administration day 2. In a small prospective randomized controlled pilot study, Blockeel et al. 18 demonstrated that the administration of corifollitropin alfa on cycle day 4 resulted in significantly reduced cycle duration and total rFSH consumption when compared with the initiation on day 2. In that study, the number of COCs obtained and ongoing pregnancy rates were comparable between the two protocols. 18 However, in the more recent study conducted by Revelli et al. 19 and apart from the comparable reproductive outcomes for normal/high responders, live birth rate/ovum pickup was significantly lower for poor responders using corifollitropin alfa on day 4, who also experienced a 40% cancellation rate due to monofollicular response. In an RCT involving women < 40 years old and fulfilling the Bologna criteria for poor ovarian response, Drakopoulos et al. 20 were not able to find differences in ongoing pregnancy rates for corifollitropin alfa followed by highly purified human menopausal gonadotrophin (hp-HMG) from stimulation day 8 onwards (investigational group), or rFSH alone (reference group). Live birth rates and the number of oocytes yielded (whether total or metaphase II) were also similar between the groups. However, more women in the corifollitropin group had exceeding embryos for cryopreservation when compared with controls. 20 21 Despite the important friendly appeal of reducing the number of injections, barriers related to the high cost of medication for ovarian stimulation in ART remain. In their small RCT, Decleer et al. 22 proposed the use of low-dose daily hCG replacing rFSH or hp-HMG, which are often more expensive. They observed a 45% cost reduction of IVF and an at least similar (but apparently higher) pregnancy rate. 22 At last, Fatemi et al. 23 conducted a small proof of concept study to evaluate if corifollitropin alfa would support the same 7-day action on follicular stimulation in a long GnRH agonist protocol (triptorelin initiated in the mid-luteal phase). Ongoing pregnancies were documented in 28 and 33.3% of started cycles for 100 μg and 150μg, respectively. Nevertheless, the duration of stimulation was ∼ 2 days longer. 23

## Is Corifollitropin Alfa an Interesting Option for Poor Responders?

Poor ovarian response to gonadotropins remains one of the greatest challenges in clinical practice, and corifollitropin alfa has been raised as an interesting option because of its longer half-life and the faster achievement of the circulating FSH threshold. The first RCT to evaluate the use of corifollitropin alfa in poor responders (defined as a previous retrieval of ≤ 4 COCs in a previous IVF cycle) was conducted by Kolibianakis et al. 24 and demonstrated that the long-acting gonadotropin is not inferior to high doses of daily follitropin beta, regarding the number of COCs retrieved. Clinical pregnancy and live birth rates were both higher among corifollitropin alfa users, but statistical analysis was not able to determine significant differences. It is important to say that results were shown to be unchanged after conforming patients to the Bologna criteria for poor ovarian response. 24 In the study by Taronger et al., 25 no differences were found for ongoing pregnancy rate, live birth rate, and the cumulative live birth rate per started cycle in potential poor responders using corifollitropin alfa plus hp-HMG or daily hp-HMG. Authors were not able to probe noninferiority of one to the other. 25 Fusi et al. 26 have recently tested corifollitropin alfa for women presenting at least 2 criteria of the following: antral follicle count (AFC) < 5, anti-Müllerian hormone (AMH) < 1.1 ng/mL, < 3 oocytes yielded in a previous cycle, and age > 40 years old. They obtained significantly higher number of retrieved oocytes and pregnancy rates, especially for women undergoing a long agonist protocol with triptorelin. Finally, in this study, shorter duration of ovarian stimulation and less cycles cancelled were attributed to corifollitropin alfa when compared with daily gonadotropins. 26 As previously demonstrated, body weight seems to remain the major determinant of exposure to corifollitropin alfa and reproductive results. 27 New information on the pharmacokinetic profile of corifollitropin alfa has been added since the original studies. Zandvliet et al. 28 retrospectively analyzed data from phase II and III clinical trials and maintained the recommendation of dosing corifollitropin alfa according to body weight in women ≤ 36 years old. However, in account to the decrease in ovarian reserve with age, a fixed 150 μg dose has been proposed for women aged ≥ 35 years old, irrespective of body weight. 28 29

## Economic Impact

According to the study of Cruz et al., 30 the use of corifollitropin alfa by oocyte donors ( *n* = 208) increased treatment overall costs as much as the cost per oocyte yielded, nevertheless without statistical significance when compared to rFSH or hp-HMG. Similar conclusions came from the cost-effectiveness analysis conducted by Khoa et al., 31 for whom the mean total cost per patient in a single treatment cycle was higher for corifollitropin alfa ( *n* = 195) compared with rFSH ( *n* = 199), as well as the cost per live birth, in women aged 35 to 42 years old weighing ≥ 50 kg undergoing IVF or intracytoplasmic sperm injection (ICSI). In contrast, Barrenetxea et al., 4 in their more robust cost-minimization analysis involving 1,390 women aged 35 to 42 years old from the PURSUE study, found a saving of ∼ 20% in the final pharmacological cost of treatment cycles using corifollitropin alfa compared with rFSH. 4 However, despite the fact that more reliable analyses can be obtained from larger studies, it seems that current available data is insufficient to offer definite conclusions on the economic benefit of one ovarian stimulation strategy over another.

Even though corifollitropin alfa has been introduced more than a decade ago, and despite the reported greater satisfaction using the long-acting gonadotropin, 32 it seems to remain less used than daily rFSH. Providing similar ongoing pregnancy rates compared with those observed for daily gonadotropins, the single injection of corifollitropin alfa offers an attractive patient-friendly option for women undergoing ovarian stimulation for ART, even though a GnRH antagonist daily injection from mid-follicular phase is still needed, as well as final trigger injection. In addition to the comfort of more injection-free days, it possibly reduces financial costs, psychological distress, and treatment drop-out. There is also an opportunity to improve patient-friendliness of ovarian stimulation for IVF by offering a needle-free option for premature luteinizing hormone surge prevention. In that way, progestin-primed protocols have been studied in the last years, including oral medroxyprogesterone acetate or dydrogesterone, with satisfactory outcomes, but limited to freeze-all cycles. 33 34 35 36 Women at risk for a hyper-response, such as those with a history of OHSS, with polycystic ovary syndrome (PCOS) or with a high antral follicle count (> 19), remain to compose the group for whom corifollitropin alfa should be avoided. 3 Nevertheless, small observational studies have been demonstrating the feasibility of corifollitropin alfa use in PCOS, if the GnRH antagonist protocol is combined with GnRH agonist triggering and total embryo cryopreservation. 37

## Conclusion

Finally, regarding patient-friendly approaches, there are alternative paths for corifollitropin alfa to be tested, such as overlapping doses, with the second dose being administered on the 4 ^th^ or 5 ^th^ day of stimulation, when the fall in circulating levels could justify a new follicular boost. Aiming at greater comfort due to the eventual achievement of the desired follicular response with only two gonadotropin applications, poor responders could be a safe target audience to be evaluated in future RCTs.
